# Supplementary material for: Interventions to Improve Medication Adherence in Patients with Schizophrenia or Bipolar Disorders: A Systematic Review and Meta-Analysis
Source: Int J Environ Res Public Health. 2021 Sep 28;18(19):10213. doi: 10.3390/ijerph181910213 (PMC8508496; doi:10.3390/ijerph181910213)
Supplement: Supplementary file 1 [file ijerph-18-10213-s001.zip › ijerph-1326808-supp-final.pdf]

International Journal of
Environmental Research and Public Health

MDPI

Table S1. Characteristics of included studies. Aho-Mustonen (2010) [52]

Methods	Study design: Exploratory RCT<br>Methods of randomisation: Block randomisation.<br>Follow-up: Baseline and 3 months post treatment.<br>Setting: An inpatient psychiatric hospital in Finland.<br>Date it was conducted: Participants were recruited in January 2006. No specific information.<br>Source of funding: Not reported.<br>Conflict of interest: Not reported.	
Participants	Inclusion/exclusion criteria: All forensic patients with a primary diagnosis of schizophrenia of schizoaffective disorder were candidates for inclusion. Exclusion criteria were evidence of organic brain syndrome, primary diagnosis of delusional disorder and earlier participation in a psychoeducational group.<br>Sample size: 39 (IG=19, TAU=20)<br>Gender: 35 (90%) were men.<br>Age: The mean age was 38.6 years (SD 14.0) in the intervention group and 40.6 (SD 8.5) in the control group.	
Interventions	Type of intervention: Educational.<br>The psychoeducation programme (intervention group) consisted of 8 group sessions which were conducted once a week; they were 45-50 minutes long (3-8 participants in each group).	
Outcomes	Primary outcome measured:<br>(1) knowledge, (2) insight illness, (3) adherence, (4) drug attitude, (5) symptoms of mental disorder, (6) ward behaviour, (7) self-reported depressive symptoms, (8) self-esteem, (9) quality of life, (10) stigma<br><br>The nursing staff assessed adherence at post-treatment and 3-month follow-up with the Compliance Rating Scale.	
**Risk of bias**		
**Bias**	**Authors' judgement**	**Support for judgement**
Random sequence generation (selection bias)	Low risk	Block randomisation was reported.
Allocation concealment (selection bias)	High risk	Patients were specifically asked not to tell the interviewer anything about their group allocation but two patients in condition did.
Blinding of participants and personnel (performance bias) all outcomes	High risk	Blinding of patients was not possible.<br>Data collection blinding failed.
Blinding of outcome assessment (detection bias) all outcomes	Unclear risk	Insufficient information to permit clear judgement.
Incomplete outcome data (attrition bias) all outcomes	Low risk	No missing outcome data are reported. Three patients dropped out at 3 months follow-up. ITT analysis was performed.
Selective reporting (reporting bias)	Unclear risk	The study protocol is not available. No adherence results were found in the text → only in a table.
Other bias	High risk	The risk may be explained by limited follow-up. Self-reported responses can be affected by

		desirability biases and the small sample size could increase the likelihood of a type II error and other bias.
Awan Riaz (2017)[44]		
Methods	Study design: RCT<br>Methods of randomisation: Randomisation was done using a computer-generated method.<br>Follow-up: Baseline and 3 months.<br>Setting: Inpatient psychiatric hospital in Pakistan.<br>Date it was conducted: February 2015 – August 2015.<br>Source of funding: Not reported.<br>Conflict of interest: Not reported.	
Participants	Inclusion/exclusion criteria: Patients who fulfilled the ICD-10 diagnostic criteria for schizophrenia were included. Patients who were not able to respond or communicate and with any other psychiatric co morbidity like severe psychical problem were excluded.<br>Sample size: 103 patients were recruited: 53 in the intervention group and 50 in the control group.<br>Gender: 80 (78%) were men.<br>Age: The mean age was 30.6 years (SD 9.5) in the intervention group and 30.4 (SD 9.4) in the control group.	
Interventions	Type of intervention: Educational.<br>One session/month; during 3 months.<br>The intervention had four parts, first part was about giving simple explanations of possible causal factors, second section focused on the nature of schizophrenia describing common symptoms and behaviours in terms of thinking, feelings and behaviour. The third section described the function of the relevant psychiatric services and the role of neuroleptic medication, fourth section was concerned with helping relatives to identify support services in terms of hospital and community resources available.<br>The control group received the treatment provided by the psychiatrist in routine clinical care (antipsychotic medication).	
Outcomes	Primary outcome measured: Adherence.<br>The Compliance Rating Scale was administered on baseline and on three-month follow-up to check the patient's adherence to treatment.	
**Risk of bias**		
**Bias**	**Authors' judgement**	**Support for judgement**
Random sequence generation (selection bias)	Low risk	Computer-generated randomisation was reported.
Allocation concealment (selection bias)	Unclear risk	No information on concealment was reported.
Blinding of participants and personnel (performance bias) all outcomes	Unclear risk	No information on blinding was reported.
Blinding of outcome assessment (detection bias) all outcomes	Unclear risk	No information on blinding was reported.
Incomplete outcome data (attrition bias) all outcomes	High risk	Missing outcome data were reported. Seven participants were lost to follow-up in the intervention group and 14 participants in the control group. No information on dealing with missing data strategies was reported.

Selective reporting (reporting bias)	Unclear risk	The study protocol is not available. Limited results related to adherence.
Other bias	High risk	The risk may be explained by limited follow-up, self-reported responses can be affected by desirability biases and gender bias.
Barkhof (2013) [53]		
Methods	Study design: RCT<br>Methods of randomisation: Randomisation was done using a computer-generated cluster method.<br>Follow-up: Baseline, 6 and 12 months.<br>Setting: In- and outpatients in three mental health care institutions in Amsterdam.<br>Date it was conducted: 2012<br>Source of funding: Dr. Paul Janssen Foundation.<br>Conflict of interest: Not reported.	
Participants	Inclusion/exclusion criteria: All patients with a primary diagnosis of schizophrenia of schizoaffective disorder, an age of 18-65 years, experienced a recent (<1 year) psychotic relapse and/or a clinical deterioration, both the following nonadherence to antipsychotic treatment resulting in hospitalisation were candidates for inclusion.<br>Exclusion criteria were an organic disease with a possible etiological relation to the psychotic disorder and/or a severe intellectual dysfunction.<br>Sample size: 114 patients were recruited: 55 in the motivational interviewing group and 59 in the health education group.<br>Gender: 91 (80%) were men.<br>Age: The mean age was 37 years (SD 1.4) in the motivational interviewing group and 34.7 (SD 1.4) in the health education group.	
Interventions	Type of intervention: Behavioural versus educational.<br>Motivational interviewing comprised 4 phases. The phases involved introduction and engagement; exploring attitudes and beliefs toward treatment, exploring patient's own personal goals and the "readiness for change". In the next phase, information was provided and ambivalences were amplified along which favourable attitudes and beliefs toward change were reinforced. The last phase was committed to evaluation and consolidation of the motivation to change.<br>Health education comprised individual lectures on general health topics like food and physical exercise.<br>Within a period of 26 weeks, participants were offered eight sessions of either motivation interviewing of health education. Less than five sessions were counted as a dropout. The sessions duration varied between 20 and 45 minutes.	
Outcomes	Primary outcome measured: Adherence.<br>Medication adherence was assessed with the Medication Adherence Questionnaire (MAQ). Before starting the intervention, a baseline assessment was performed. Participants were interviewed again after the intervention was completed and after six-month follow-up.	
**Risk of bias**		
**Bias**	**Authors' judgement**	**Support for judgement**
Random sequence generation (selection bias)	Low risk	Computer-generated cluster randomisation with blocks of codes for every 6 consecutive inclusions which were 1 by 1 revealed to the study coordinator was reported.
Allocation concealment (selection bias)	Low risk	Participants were allocated to either the motivational interviewing or the health education group by a computerised cluster randomisation program which were one by one

		revealed by the coordinating researcher. Patients were not informed about the intervention groups.
Blinding of participants and personnel (performance bias) all outcomes	Low risk	The psychologists, psychiatrics and community health nurses were blinded.
Blinding of outcome assessment (detection bias) all outcomes	Low risk	The assessors were blinded.
Incomplete outcome data (attrition bias) all outcomes	High risk	Missing outcome data were reported and were likely to be related to true outcome.
Selective reporting (reporting bias)	Low risk	The study protocol is not available but the authors are transparent in the abstract, results, discussion and conclusion concerning the results.
Other bias	High risk	Self-reported responses can be affected by desirability biases.
Bäuml (2016) [54]		
Methods	Study design: RCT<br>Methods of randomisation: Block randomisation.<br>Follow-up: 24 months and 84 months.<br>Setting: Three psychiatric hospitals in Munich, Germany.<br>Date it was conducted: 1990 - 1994.<br>Source of funding: The first two years of the study were supported by a grant from the Bundesministerium für Forschung und Technologie (BMFT); the long-term follow-up was supported by a grant from the DORIST- Fond, in Kreuzlingen, Switzerland.<br>Conflict of interest: Not reported.	
Participants	Inclusion/exclusion criteria:<br>The inclusion criteria were patients with a schizophrenic or schizoaffective disorder (DSM III-R: 295.10-94; 297.10/ International Classification of Diseases (ICD)-10: F 20, F22, F25), an indication of antipsychotic relapse prevention for a period of at least 12 months, age between 18 and 65 years, patients' acceptance of an outpatient treatment in the study centre and patients' agreement to involve a key relative or a friend.<br>*Exclusion Criteria were* a distance between home and hospital of more than 150 kilometres, less than 30 minutes contact per week with the key relative, drug addiction during the past six months prior to admis- sion, pregnancy, IQ < 80, insufficient knowledge of the German language and no remission of the psychotic symptoms during the last two years despite a sufficient therapy.<br>Sample size: 41 (IG=21, TAU=20).<br>Gender: In the intervention group were 48% men and in the control group 35%.<br>Age: The mean age was 38 years (SD 7.9) in the intervention group and 41 (SD 9.4) in control group.	
Interventions	Type of intervention: Educational.<br>There were 4 weekly sessions of 60 minutes each; afterwards, 4 additional monthly sessions were held. Relatives were also invited to 8 weekly sessions, each lasting 90 minutes. The groups were headed by therapists who had not been involved in the routine treatment. In both settings the same psychoeducational modules were presented. Apart from improvement of coping by discussing similar experiences, considerable	

	attention was paid to the interactive evaluation of illness relevant information. The take-home message of the psychoeducational program was: schizophrenic psychoses are provoked by biological factors in combination with psychosocial stress; therefore, they have to be treated with medication and psychotherapeutic interventions. Patients' empowerment can only be developed successfully on the basis of a sufficient medication and long-term psychosocial treatment elements. Above all, the patients were trained to report their side effects to their therapists immediately and to look together with them for the most suitable medication.	
Outcomes	Primary outcome measured:<br>(1) adherence; (2) type of medication; (3) mean number of consumed CPZ-units; (4) neuroleptic side effects of medication.<br><br>Adherence was rated by the treating psychiatrists on a four-step ordinal scale (1 = very good/ 2 = good/ 3 = moderate/ 4 = bad). Plasma drug level measurements were performed in order to validate the psychiatrists' adherence ratings.	
**Risk of bias**		
**Bias**	**Authors' judgement**	**Support for judgement**
Random sequence generation (selection bias)	Low risk	Block randomisation was reported. The randomisation list was generated by computerised random sampling.
Allocation concealment (selection bias)	Unclear risk	No information on concealment was reported.
Blinding of participants and personnel (performance bias) all outcomes	Unclear risk	No information on blinding was reported.
Blinding of outcome assessment (detection bias) all outcomes	Unclear risk	No information on blinding was reported.
Incomplete outcome data (attrition bias) all outcomes	High risk	Missing outcome data were high (60 patients dropped out) and were likely to be related to true outcome.
Selective reporting (reporting bias)	Low risk	The study protocol is not available but it is clear that the published reports include all expected outcomes.
Other bias	Low risk	The small sample size could increase the likelihood of a type II error.
Beebe (2014) [61]		
Methods	Study design: RCT<br>Methods of randomisation: Ad random (using a table of random numbers to one of three groups).<br>Follow-up: Baseline and 3 months.<br>Setting: An outpatient community mental health centre in the Southeastern United States.<br>Date it was conducted: Not reported.<br>Source of funding: Not reported.<br>Conflict of interest: The authors report no conflicts of interest.	
Participants	Inclusion/exclusion criteria:<br>Inclusion criteria were age between 21-68 years, receiving outpatient care, chart diagnosis of schizophrenia or schizoaffective disorder and English speaking.	

	Exclusion criteria were chart documentation of mental retardation or developmental delay, hearing loss prohibiting telephone communication or vision or dexterity problems prohibiting texting.<br>Sample size: 30.<br>Gender: 11 (37%) were men.<br>Age: The mean age was 48.7 years (SD 11.6).	
Interventions	Type of intervention: Behavioural (electronic interventions).<br>Three intervention arms: (1) weekly telephone-intervention only; (2) daily text messages only and (3) combination of weekly telephone interventions and daily text messages<br>1. Telephone call intervention: A weekly telephone call during three months with problem solving strategies (medication, appointments, symptoms, cravings) to provide weekly support.<br>2. Texting intervention: The participants in this group received a daily text message for three months. The topics are the same as the telephone call intervention.<br>3. Combined telephone intervention and texting intervention: Participants in this group received weekly phone calls and daily text messages as described in the telephone call and texting intervention for three months.	
Outcomes	Primary outcome measured: Adherence.<br>A measure of adherence was generated by pill counts.	
**Risk of bias**		
**Bias**	**Authors' judgement**	**Support for judgement**
Random sequence generation (selection bias)	Low risk	Participants were randomly assigned (using a table of random numbers) to one of the three groups.
Allocation concealment (selection bias)	High risk	The principal investigator was blinded as to group assignment when conducting the baseline assessment. Afterwards the principal investigator was aware of the allocation because he was responsible for performing the intervention.
Blinding of participants and personnel (performance bias) all outcomes	Unclear risk	Insufficient information to permit clear judgement.
Blinding of outcome assessment (detection bias) all outcomes	Unclear risk	Insufficient information to permit clear judgement.
Incomplete outcome data (attrition bias) all outcomes	Low risk	Only 2 patients dropped out during the three months follow-up.
Selective reporting (reporting bias)	Unclear risk	The study protocol is not available but the non-significant results were minimized and only the beneficial results were showed.
Other bias	High risk	The small sample size and low power could increase the likelihood of a type II error. The risk also may be explained by limited follow up.
Beebe (2016) [19]		
Methods	Study design: RCT<br>Methods of randomisation: Convenience sample.<br>Follow-up: Baseline and 3 months.<br>Setting: An outpatient community mental health centre in the Southeastern United States.<br>Date it was conducted: Not reported.	

	Source of funding: Agency for Healthcare Research and Quality.<br>Conflict of interest: The authors report no conflicts of interest.	
Participants	Inclusion/exclusion criteria:<br>Inclusion criteria were chart diagnosis of schizophrenia or schizoaffective disorder, not hospitalised for psychiatric illness within the past six months and English speaking.<br>Exclusion criteria were a chart of diagnosis of coexisting mental retardation, neurological disorders or head injury.<br>Sample size: 140<br>Gender: 80 (57%) were men.<br>Age: The mean age was 46.1 years (SD 12.9).	
Interventions	Type of intervention: Behavioural (electronic interventions).<br>Telephone call intervention: A weekly telephone call during three months with problem solving strategies (medication, appointments, symptoms, cravings) to provide weekly support.	
Outcomes	Primary outcome measured:<br>(1) Medication adherence; (2) Schizophrenia symptoms<br><br>The Medication Adherence Rating Scale was used to measure self-reported medication adherence.	
**Risk of bias**		
**Bias**	**Authors' judgement**	**Support for judgement**
Random sequence generation (selection bias)	Unclear risk	Insufficient information to permit clear judgement.
Allocation concealment (selection bias)	Unclear risk	No information on concealment was reported.
Blinding of participants and personnel (performance bias) all outcomes	Unclear risk	No information on blinding was reported.
Blinding of outcome assessment (detection bias) all outcomes	Unclear risk	No information on blinding was reported.
Incomplete outcome data (attrition bias) all outcomes	Unclear risk	No missing outcome data are reported.
Selective reporting (reporting bias)	Unclear risk	Insufficient information to permit judgement due to a lack of details provided on the methodology and results.
Other bias	High risk	The risk may be explained by limited follow-up. Self-reported responses can be affected by desirability biases.
Çetin (2018) [55]		
Methods	Study design: RCT with pre and post-test<br>Methods of randomisation: A simple random sampling.<br>Follow-up: Not reported.<br>Setting: Community Mental Health Centres (CMHC) located in Balıkesir and Eskişehir provincial centres, Turkey.<br>Date it was conducted: February 2016 – May 2016.<br>Source of funding: Not reported.<br>Conflict of interest: Not reported.	

Participants	Inclusion/exclusion criteria:<br>The inclusion criteria were to be between 18 and 65 years of age, to be literate, to be open to communication and cooperation, to have been diagnosed with schizophrenia according to DSM-IV criteria for the last one year.<br>Patients with acute exacerbations, active alcohol or psychoactive substance use, mental condition which makes impossible the communication and cooperation like mental retardation or de- mentia were excluded from the study.<br>Sample size: 135 (IG=55, TAU=80).<br>Gender: In the intervention group were 67% men and in the control group 69%.
Interventions	Type of intervention: Educational<br>A total of 8 sessions were held twice a week on Mondays and Fridays so that home works and exercises could be done by the participants in the psychoeducation program. A total of 55 experimental group patients was divided into 8–12 people for psycho-education groups according to the session configuration of mindfulness therapy. Throughout the entire program, patients were provided with the opportunity to participate in psycho-therapy interactively in the form of questions and answers. Each session was held for a total of 70 min with a break of 10 min, divided into 2 with a 30 min interval taking into account the situation of the patients. During the entire psychoeducation program and after each session, counselling and support were provided by interviewing patients who had additional questions.<br>In the study, the meditation techniques of the Mindfulness Therapy constituted the backbone/framework of the psychoeducation program and were used as a means to increase insight and medication adherence in patients. Body and breath, body scanning, mindfulness movement and three-minute respiration techniques were practiced during the psychoeducation program, practically every session in accordance with the researchers' directives. The body, breath, and three-minute respiration meditation were practiced while the patient was seated on the chair with comfortable clothes, the body scanning and mindfulness movement was performed on the yoga mattress. They were also asked to perform these techniques in the form of homework at home through a meditation CDs distributed by the researcher. By using these meditation techniques, it was aimed that the patients are able to focus their attention on the present moment, to observe their own experiences, bodies, emotions and thoughts internally, to behave unprejudiced and leisurely, to accept themselves as they are, to discover their own physical and spiritual boundaries, to recognize and describe the symptoms, process, treatment and effects on their lives of disease, and develop their ability to cope with the disease. In this way, it was aimed to in- crease the insight and medication adherence of the schizophrenic patients.
Outcomes	Primary outcome measured:<br>(1) Insight; (2) Medication adherence<br><br>The Morisky and Medication Adherence Scale were used to measure self-reported medication adherence.

Risk of bias

Bias	Authors' judgement	Support for judgement
Random sequence generation (selection bias)	High risk	A simple randomisation but not random allocation was reported.

Allocation concealment (selection bias)	Unclear risk	No information on concealment was reported.
Blinding of participants and personnel (performance bias) all outcomes	Unclear risk	No information on blinding was reported.
Blinding of outcome assessment (detection bias) all outcomes	Unclear risk	No information on blinding was reported.
Incomplete outcome data (attrition bias) all outcomes	High risk	Missing outcome data were reported and were likely to be related to true outcome. High drop-out rates and no information concerning how to deal with missing data.
Selective reporting (reporting bias)	Low risk	The study protocol is not available but it is clear that the published reports include all expected outcomes.
Other bias	High risk	The risk may be explained by the unclear follow-up and the self-reported assessment tool.
Chien Tong (2015) [45]		
Methods	Study design: RCT<br>Methods of randomisation: A set of computer-generated random numbers provided by an independent statistician.<br>Follow-up: Baseline, immediately post intervention and 6 months post intervention.<br>Setting: One Community Psychiatric Nursing Service.<br>Date it was conducted: December 2012 – January 2014.<br>Source of funding: Financial support by the Health and Medical Research Fund, Food and Health Bureau, the Government of Hong Kong.<br>Conflict of interest: Not reported.	
Participants	Inclusion/exclusion criteria:<br>Patients were included if they were aged between 18 and 60 years, Hong Kong residents speaking in Mandarin or Cantonese, having a primary diagnosis of schizophrenia in the past five years and had poor adherence to medication.<br>Exclusion criteria were those patients who had regular depot or intramuscular injections only, co-morbidities of learning disability, organic brain disease and/or cognitive impairments, previous participation in any medication management program and/or hostel residents supervised by mental health workers to take their medication.<br>Sample size: 114 (IG=57, TAU=57).<br>Gender: In the intervention group were 51% men and in the control group 53%.<br>Age: The participants had a mean age of 28 to 29 years (range 18-49).	
Interventions	Type of intervention: Behavioural.<br>Motivational interviewing techniques concerning cognitive, motivational, insight inducing and behavioural training in 8 sessions during a four-month program. The first phase (two sessions) aimed to engage participants in addressing their needs for, and concerns with medication adherence, facilitating goal and action setting for changes in medication adherence. The second phase (three sessions) focused on education about the mental illness and its treatment and, then explored participants' strengths and barriers to adherence, assisting them in recognising social stigma and family support, and developing coping strategies in medication management over months. The third phase (three sessions) aimed to	

	rationalize patient's beliefs and concerns, manage their perceived of experienced social stigma, and enhance family and social support networks, thus improving relapse prevention and integration into the community.	
Outcomes	Primary outcome measured:<br>(1) Medication adherence; (2) Symptom severity; (3) Insight into treatment; (4) Hospitalisation rate; (5) Functioning<br><br>It is not clear which instrument was used. They reported only the measurement concerns a self-reported five-point Likert scale. The questions were based on the MARS, MAQ, DAI and CRS but it is unclear which questions were used.	
**Risk of bias**		
**Bias**	**Authors' judgement**	**Support for judgement**
Random sequence generation (selection bias)	Low risk	Computer-generated randomisation was reported.
Allocation concealment (selection bias)	Low risk	Participants were allocated to either the motivational interviewing or the TAU group by a computerised cluster randomisation program which were one by one revealed by an independent statistician, who was blind to the patient list.
Blinding of participants and personnel (performance bias) all outcomes	Low risk	The psychiatrists and assessing nurses were blinded.
Blinding of outcome assessment (detection bias) all outcomes	Low risk	Research assistants and community nurses were blinded for outcome assessments.
Incomplete outcome data (attrition bias) all outcomes	Low risk	The attrition rate was 3.5% and balanced in numbers across groups with similar reasons for missing data. ITT analysis was performed.
Selective reporting (reporting bias)	Low risk	The study protocol is not available but it is clear that the published reports include all expected outcomes.
Other bias	Low risk	The risk may be explained by the unclear assessment tool.
Dahan (2016) [46]		
Methods	Study design: RCT<br>Methods of randomisation: Randomly assigned via lottery drawing.<br>Follow-up: After the intervention.<br>Setting: An active open unit in a Mental Health Centre in Tel-Aviv.<br>Date it was conducted: January 2009 and April 2010.<br>Source of funding: Not reported.<br>Conflict of interest: Not reported.	
Participants	Inclusion/exclusion criteria:<br>Hospitalised patients diagnosed with schizophrenia and aged between 18 and 60 years.<br>Sample size: 63 (IG=31, TAU=32).<br>Gender: Twenty-four (80%) were men in each group.<br>Age: The mean age was 36.1 years (SD 8.9) in the intervention group and 39.67 (SD 10.6) in control group.	
Interventions	Type of intervention: Mixed<br>The intervention combined psycho-education, cognitive-behavioural	

	strategies and motivational interviewing.<br><br>Each participant in the intervention group attended an average of 6 sessions spread over once to twice a week and lasting approximately 20-40 minutes. The sessions were one on one with the same nurse.<br><br>1. The psycho-education aimed to promote understanding of the disease process and improve attitude toward treatment.<br>2. The cognitive-behavioural strategies aimed problem solving techniques for increasing attention and decreasing forgetfulness.<br>3. Motivational interviewing aimed at exploring the patient's perspective on the illness and placing it into a coherent life narrative.	
Outcomes	Primary outcome measured:<br>(1) Medication adherence; (2) Drug attitude inventory<br><br>The Visual Analog Scale for Assessing Treatment Compliance was used to measure self-reported medication adherence.	
**Risk of bias**		
**Bias**	**Authors' judgement**	**Support for judgement**
Random sequence generation (selection bias)	High risk	Randomly assigned via lottery drawing was reported. It was unclear how the lottery drawing was done.
Allocation concealment (selection bias)	Unclear risk	No information on concealment was reported.
Blinding of participants and personnel (performance bias) all outcomes	Unclear risk	No information on blinding was reported.
Blinding of outcome assessment (detection bias) all outcomes	Unclear risk	No information on blinding was reported.
Incomplete outcome data (attrition bias) all outcomes	Low risk	There was no drop-out.
Selective reporting (reporting bias)	Low risk	The study protocol is not available but it is clear that the published reports include all expected outcomes.
Other bias	High risk	The risk may be explained by the unclear follow-up. Self-reported responses can be affected by desirability biases.
Eker (2012) [56]		
Methods	Study design: Semi-experimental study, pre and post-test with IG and CG.<br>Methods of randomisation: Not reported.<br>Follow-up: 2,5 months.<br>Setting: University Hospital Mood Disorders Outpatient Clinic in Turkey.<br>Date it was conducted: April 2009 – May 2009.<br>Source of funding: No funding.<br>Conflict of interest: No conflicts of interest.	
Participants	Inclusion/exclusion criteria:<br>Patients were included if they were having the diagnosis of Bipolar Affective Disorder, were able to learn the defined concepts in every learning activity and would stay calmly during the sessions.<br>Sample size: 71 (IG=36, TAU=35).<br>Gender: In the intervention group were 46% men and in the control group	

	47%.<br>Age: The mean age was 34.6 years (SD 11.3) in the intervention group and 36.64 (SD 10.6) in control group.	
Interventions	Type of intervention: Educational.<br>The psycho-education program consisted of six sessions lasted 90-120 minutes, groups of 10-12 persons and were held once a week. In every session, learning objectives and aims were stated: interactive teaching methods like role playing, question and answers, discussion and presentation.	
Outcomes	Primary outcome measured: Adherence.<br>The Medication Adherence Rating Scale was used to measure self-reported medication adherence.	
**Risk of bias**		
**Bias**	**Authors' judgement**	**Support for judgement**
Random sequence generation (selection bias)	Unclear risk	No information on randomisation was reported.
Allocation concealment (selection bias)	Unclear risk	No information on concealment was reported.
Blinding of participants and personnel (performance bias) all outcomes	Unclear risk	No information on blinding was reported.
Blinding of outcome assessment (detection bias) all outcomes	Unclear risk	No information on blinding was reported.
Incomplete outcome data (attrition bias) all outcomes	Low risk	The drop-out rates were 5 patients in the IG and 3 patients in the CG. Two types of analysis were performed (1) analysis of completers only; (2) last observation carried forward (LOCF).
Selective reporting (reporting bias)	Unclear risk	The study protocol is not available and not much results details provided.
Other bias	High risk	The risk may be explained by limited follow-up. Self-reported responses can be affected by desirability biases.
Ertem (2018) [21]		
Methods	Study design: RCT<br>Methods of randomisation: Randomisation (simple numbers table).<br>Follow-up: Baseline, immediately post intervention, 3 and 6 months follow-up.<br>Setting: University hospital psychiatry outpatient clinic in Turkey<br>Date it was conducted: December 2014 – October 2015.<br>Source of funding: No funding.<br>Conflict of interest: There is no conflict of interest between the authors.	
Participants	Inclusion/exclusion criteria:<br>Patients were included if they were aged between 18 and 65 years, the diagnosis of schizophrenia, able to read and write Turkish, as were willing and able to be interviewed.<br><br>Exclusion criteria were history of chronic physical disease, a history of substance use (except caffeine and nicotine) and a history of mental retardation.<br>Sample size: 40<br>Gender: In the intervention group were 70% men and in the control group	

	50%.<br>Age: The mean age was 43.2 years (SD 10.5) in the intervention group and 40.1 (SD 10.9) in control group.	
Interventions	Type of intervention: Behavioural.<br>The intervention program consists of 6 semi-structured, interconnected interviews. All the interviews were interconnected with themselves because of providing topic integrity. Each interview lasted 40-60 minutes on average and the process was completed in a total of 6 by weekly interviews.	
Outcomes	Primary outcome measured: Adherence.<br>The Morisky scale was used to measure self-reported medication adherence.	
**Risk of bias**		
**Bias**	**Authors' judgement**	**Support for judgement**
Random sequence generation (selection bias)	Low risk	Randomisation (simple numbers table) was reported.
Allocation concealment (selection bias)	Unclear risk	No information on concealment was reported.
Blinding of participants and personnel (performance bias) all outcomes	Unclear risk	No information on blinding was reported.
Blinding of outcome assessment (detection bias) all outcomes	Unclear risk	No information on blinding was reported.
Incomplete outcome data (attrition bias) all outcomes	Low risk	No missing outcome data are reported. ITT analysis were performed.
Selective reporting (reporting bias)	High risk	The study protocol is not available and risk for multiple testing.
Other bias	Low risk	Self-reported responses can be affected by desirability biases.
Guo (2010) [47]		
Methods	Study design: RCT<br>Methods of randomisation: 1:1 randomisation.<br>Follow-up: 12 months.<br>Setting: 10 clinical outpatient psychiatric clinics in China.<br>Date it was conducted: 1 January 2005 – 31 October 2007.<br>Source of funding: National Key Technologies R&D Program of China and National Natural Science Foundation of China.<br>Conflict of interest: Funding organizations played no role in the design, conduct, analysis or interpretation of the research in any aspect of preparation or approval of the manuscript.	
Participants	Inclusion/exclusion criteria:<br>The inclusion criteria were aged 18 to 50 years, a diagnosis of schizophrenia or schizoaffective disorder within the past five years, living with family members who could be involved in the patient's care, Positive and Negative Syndrome Scale total score of 60 or less, receiving maintenance treatment with one antipsychotics.<br><br>Patients were excluded if they were prescribed two or more antipsychotics or long-acting injectable antipsychotics, participating in other therapy programs, pregnant or diagnosed as having a serious and unstable medical	

	condition.<br>Sample size: 1268 (IG=633, TAU=635).<br>Gender: In the intervention group were 344 (54%) men and in the control group 354 (56%).<br>Age: The mean age was 26.1 years in the intervention group and 26.4 in control group.	
Interventions	Type of intervention: Mixed.<br>The intervention consists of psycho-education, family intervention, skills training and cognitive behaviour therapy administered during 48 group sessions. Participants were seen 12 times (once per month for 12 months), receiving each of the 4 group treatments on the same day, for a total of 48 one-hour sessions.	
Outcomes	Primary outcome measured:<br>(1) Relapse<br>Secondary outcome measured:<br>(1) Insight; (2) medication adherence; (3) Quality of life; (4) Social functioning.<br><br>The psychiatrists assessed participants monthly for medication adherence on appointment adherence. It is unclear which instrument was used.	
**Risk of bias**		
**Bias**	**Authors' judgement**	**Support for judgement**
Random sequence generation (selection bias)	Unclear risk	Insufficient information to permit clear judgement.
Allocation concealment (selection bias)	Unclear risk	No information on concealment was reported. ((cfr. to be checked later in paper of 2007, not accessible on 16/12/2019).
Blinding of participants and personnel (performance bias) all outcomes	Low risk	The clinicians were blinded.
Blinding of outcome assessment (detection bias) all outcomes	Low risk	The assessors were blinded.
Incomplete outcome data (attrition bias) all outcomes	High risk	High drop-out rates. Only 60% of patients completed one year follow-up. No information provided about dealing with missing data.
Selective reporting (reporting bias)	Unclear risk	The study protocol is not available and raw data results concerning the intervention improved adherence were unclear.
Other bias	Low risk	The risk may be explained by the unclear assessment tool.
Javadpour (2013) [48]		
Methods	Study design: RCT<br>Methods of randomisation: Randomly with equal sets of odd and even numbers in a sealed envelope and send to the researcher.<br>Follow-up: 18 months (baseline, 6-8-12 months follow-up).<br>Setting: Hospital in Shiraz, Iran.<br>Date it was conducted: June 2010 – November 2011.<br>Source of funding: Shiraz University of Medical Science.<br>Conflict of interest: None declared.	
Participants	Inclusion/exclusion criteria:<br>Patients were included if they were having the diagnosis of Bipolar Affective Disorder, were aged between 18 and 60 and had a history of at	

	least two episodes of relapse in the past two or three episodes in last five years.<br>Sample size: 108 (IG=54, TAU=54).<br>Gender: In the intervention were 22 and the control group were 20 men.<br>Age: Not reported.	
Interventions	Type of intervention: Educational.<br>Participants in the intervention group received individual psycho-education. The program consisted of 8 sessions each consisting of a 50 min session per week including: understanding bipolar disorder and its aetiology, familiarisation with symptoms of mania and hypomania, understanding signs of depression and other episodes, awareness of causes and prognosis, education about the functions, types and adverse side effects of antimanic and antidepressant medications.<br>Participants also received information about the risk of discontinuation of these medications, learning how to detect any future episodes of relapse as well as strategies and plans on which to base early detection of symptoms and for being self-directed towards new situations.<br>After the sessions of face to face individual education, the intervention continued using scheduled monthly telephone contact to remind the participants of their next appointment. Each telephone contact consisted of a 10 min question and answer.	
Outcomes	Primary outcome measured:<br>(1) Quality of life; (2) Symptoms of relapse; (3) Medication adherence.<br><br>The Medication Adherence Rating Scale was used to measure self-reported medication adherence.	
**Risk of bias**		
**Bias**	**Authors' judgement**	**Support for judgement**
Random sequence generation (selection bias)	Low risk	Randomly with equal sets of odd and even numbers in a sealed envelope and send to the researcher.
Allocation concealment (selection bias)	Unclear risk	No information on concealment was reported.
Blinding of participants and personnel (performance bias) all outcomes	High risk	The psychiatry resident could not be blinded because he performed the sessions.<br><br>Blinding of patients is not possible.
Blinding of outcome assessment (detection bias) all outcomes	Low risk	The assessor was blinded.
Incomplete outcome data (attrition bias) all outcomes	High risk	High drop-out rates; 33% of the IG and 24% of the CG. No information on dealing with missing data strategies was reported.
Selective reporting (reporting bias)	Low risk	The study protocol is not available but it is clear that the published reports include all expected outcomes.
Other bias	Low risk	Self-reported responses can be affected by desirability biases.
Jones (2015) [57]		
Methods	Study design: RCT<br>Methods of randomisation: Randomised by an independent clinical trials unit.<br>Follow-up: Baseline, 6, 12 and 15 months post intervention.	

	Setting: Community mental health and outpatient clinics.<br>Date it was conducted: 9 February 2011 – 19 January 2012.<br>Source of funding: National Institute for Health Research, England.<br>Conflict of interest: Not reported.	
Participants	Inclusion/exclusion criteria:<br>Patients were included if they were having the diagnosis of Bipolar Affective Disorder with onset in past five years, were aged between 18 and 65 years, sufficient understanding of written and spoken English in order to provide consent and engage with interviews and use the intervention.<br><br>Exclusion criteria included: manic, hypomanic and depressed or mixed episode currently or in the past four weeks.<br>Sample size: 67 (IG=34, TAU=33).<br>Gender: In the intervention group 25 (76%) were female and 22 (64%) in the control group.<br>Age: The mean age was 38.3 years (SD 12.8) in the intervention group and 39.9 (SD 10.4) in the control group.	
Interventions	Type of intervention: Behavioural.<br>The intervention group received an 18 hour delivered therapy over approximately 6 months at client's homes or mental health facilities, according to personal preference.<br>Initial sessions were weekly and typically lasted 45-60 minutes.<br><br>The following elements are included: meaning and relevance of diagnosis, identification of recovery-informed therapy goals, initial formulation of relationships between mood experiences and progress towards recovery goals, identification and application of CBT techniques to address and facilitate positive coping and considering of wider functioning issues in relation to recovery.	
Outcomes	Primary outcome measured:<br>(1) Level of recruitment in the trial; (2) Retention of patients into both study arms; (3) Adherence to the intervention; (4) Completion of the intervention.<br>Secondary outcomes measured:<br>(1) Bipolar relapse; (2) Observer-rated mood; (3) Recovery; (4) Clinical measures; (5) Medication adherence.<br><br>The Stephenson Medical Adherence Questionnaire was used to measure self-reported medication adherence.	
**Risk of bias**		
**Bias**	**Authors' judgement**	**Support for judgement**
Random sequence generation (selection bias)	Low risk	Individuals were randomised by an independent clinical trials unit with minimisation on the number of previous episodes, current mood symptoms and mania, all significant predictors of therapy outcome.
Allocation concealment (selection bias)	Low risk	Participants were allocated to either groups by an independent clinical trials unit.
Blinding of participants and personnel (performance bias) all outcomes	Low risk	The clinicians, researchers and patients were blinded.

Blinding of outcome assessment (detection bias) all outcomes	Low risk	The assessors were blinded. In total, 79% of pts had masked assessments throughout and 95% of all assessment sessions were confirmed as definitely masked.
Incomplete outcome data (attrition bias) all outcomes	Low risk	Recruitment and follow-up rates within 10% of pre-planned targets for 12 months follow-up was achieved. Missing data were assumed to be missing at random (ignorable) and automatically allowed for in fitting the random-effects or analysis of covariance models
Selective reporting (reporting bias)	Low risk	The study protocol is not available but it is clear that the published reports include all expected outcomes.
Other bias	High risk	The small sample size could increase the likelihood of a type II error. The risk also may be explained to Self-reported responses can be affected by desirability biases.
Kopelowicz (2012) [43]		
Methods	Study design: RCT<br>Methods of randomisation: Randomisation was done using a computer-generated method.<br>Follow-up: Baseline, 4, 8, 12, 18 and 24 months.<br>Setting: Two community mental health centres in Los Angeles, California.<br>Date it was conducted: April 2003 – January 2007.<br>Source of funding: National Institute of Mental Health (Dr. Kopelowicz).<br>Conflict of interest: Not reported.	
Participants	Inclusion/exclusion criteria: Patients were included if they were aged between 18 and 50 years, the diagnosis of schizophrenia, spoke Spanish, had been without antipsychotic medication without medical authorisation for one continuous week in the month prior to study enrolment, lived with their family of origin and had least one family member willing to participate in the family treatment.<br>Sample size: 174 (MFG-A=64, MFG-S=53, TAU=57).<br>Gender: In the intervention groups 67% and 68% were men and in the control group 57% were men.<br>Age: Not reported.	
Interventions	Type of intervention: Educational versus mixed intervention.<br>Three arms: MFG-A (educational), MFG-S (mixed) and care as usual.<br>The MFG-A was focusing on specific obstacles to maintaining medication adherence.<br><br>The MFG-S consisted of 3 components: 3 sessions separately with each family, a one day (6 hour) multifamily educational workshops and multifamily group sessions. There were 24 sessions total spread over 12 months (twice monthly). The sessions consisted a formal discussion of the illness, discussing how schizophrenia had affected each of their lives and teaching problem-solving skills. Members were free to select any problem, regardless of its relevance to medication adherence.	
Outcomes	Primary outcome measured: Adherence.<br>The psychiatrists assessed participants monthly for medication adherence on appointment adherence with a five point Likert scale. It is unclear which instrument was used.	
**Risk of bias**		
**Bias**	**Authors' judgement**	**Support for judgement**

Random sequence generation (selection bias)	Low risk	Computer-generated randomisation was reported.
Allocation concealment (selection bias)	Unclear risk	No information on concealment was reported.
Blinding of participants and personnel (performance bias) all outcomes	Unclear risk	No information on blinding was reported.
Blinding of outcome assessment (detection bias) all outcomes	Low risk	The research assistant was blinded for the outcome assessment.
Incomplete outcome data (attrition bias) all outcomes	High risk	Missing outcome data was 26% immediately after the baseline assessments.
Selective reporting (reporting bias)	Low risk	The study protocol is not available but it is clear that the published reports include all expected outcomes.
Other bias	Low risk	The risk may be explained by the unclear assessment tool.

Menon (2018) [49]

Methods	Study design: RCT.<br>Methods of randomisation: Randomisation was done using a computer-generated method.<br>Follow-up: 3 months.<br>Setting: Department of Psychiatry of the Jawaharlal Institute of Postgraduate Medical Education and Research (JIPMER), Puducherry, India.<br>Date it was conducted: December 2015 – July 2017.<br>Source of funding: This review received no specific grant from any funding.<br>Conflict of interest: No conflict of interest has been declared by the authors.
Participants	Inclusion/exclusion criteria: The inclusion criteria were aged between 18 and 65 years, diagnosed with Bipolar I Disorder (BD-I) on the Diagnostic and Statistical Manual of Mental Disorders – fifth edition (DSM -5) (American Psychiatric Association, 2013). All patients were on a stable drug/dose regimen for at least the past one year.<br>Patients with Hamilton Depression Rating Scale (HDRS) scores ≥ 7 and Young Mania Rating Scale (YMRS) scores ≥ 8 were excluded as were patients/caregivers without access to mobile phones and patients/caregivers who were unable to read either English or the regional language (Tamil).<br>Sample size: 132 (IG=62, TAU=70).<br>Gender: In the intervention group were 55% men and in the control group 50%.<br>Age: The mean age was 37 years (SD 9.6) in the intervention group and 38.7 (SD 11.6) in control group.
Interventions	Type of intervention: Behavioural.<br>The intervention group received identical twice-weekly, text SMS reminders. The SMS messages greeted the recipient, reminded the recipient about taking medications at the times and doses prescribed, and ended with a positive message such as “Have a nice day”. During monthly follow up visits, and in the first three months of the study, intervention group

	patients were asked (by an investigator who was not involved in outcome measurement) whether they were receiving the SMS messages regularly. The TAU group received TAU alone for the entire duration of the six-month study. TAU included both pharmacologic treatment (with medications such as mood stabilizers and/or anti- psychotics) and psychosocial treatment strategies, as indicated.	
Outcomes	Primary outcome measured: Adherence.<br><br>The Morisky scale was used to measure self-reported medication adherence.<br>Secondary outcomes measured:<br>(1) Treatment attitudes; (2) Quality of life.	
**Risk of bias**		
**Bias**	**Authors' judgement**	**Support for judgement**
Random sequence generation (selection bias)	Unclear risk	Computer-generated randomisation was reported. No further details were available.
Allocation concealment (selection bias)	Unclear risk	No information on concealment was reported.
Blinding of participants and personnel (performance bias) all outcomes	Unclear risk	No information on blinding of participants and personnel was reported.
Blinding of outcome assessment (detection bias) all outcomes	Low risk	The assessors were blinded (rater-blinded assessments).
Incomplete outcome data (attrition bias) all outcomes	Low risk	By the end of the intervention phase, 16 participants had dropped out of the trial (3 and 13 in the intervention and control groups), and by the end of the subsequent 3-month follow- up phase, the cumulative study drop out was 32 (10 and 22 in the intervention and control groups). Complete data were unavailable for all study completers. ITT analyses were conducted, missing data were imputed by LOCF and sensitivity analyses examined completer samples.
Selective reporting (reporting bias)	Low risk	The study protocol is not available but it is clear that the published reports include all expected outcomes.
Other bias	Low risk	Self-reported responses can be affected by desirability biases. Power analysis was performed, including a 10% attrition/non-participation rate; estimated 60 pts per group.
Moncrieff (2016) [58]		
Methods	Study design: RCT<br>Methods of randomisation: Cluster randomisation based on an internet randomisation service (sealed envelope) using block size.<br>Follow-up: 1 and 3 months post intervention.<br>Setting: Community recovery, North East London.<br>Date it was conducted: Not reported.<br>Source of funding: National Institute for Health Research.<br>Conflict of interest: No conflict of interest.	
Participants	Inclusion/exclusion criteria:<br>Patients had to be over the age of 18, have a diagnosis of psychosis,	

		schizophrenia or schizoaffective disorder or a mood disorder with psychotic symptoms and be currently taking antipsychotic medication. Patients were required to have an allocated health professional who was usually a nurse, social worker or occupational therapist from the participant's clinical team. They also needed to have a consultation with their psychiatrist pending within the next three months.<br><br>Patients who could not speak English or lacked capacity to consent were excluded from the study.<br>Sample size: 60 (IG=31, TAU=29).<br>Gender: In the intervention group were 74% men and in the control group 69.<br>Age: The mean age was 45 years in the intervention group and 39 in control group.
Interventions		Type of intervention: Educational.<br>The Medication Review Tool and website was designed to provide information about psychotic conditions including schizophrenia, types of antipsychotic medication and points for people to consider when discussing and making decisions about medication with professionals. It included links to external sites for users to access more detailed information.
Outcomes		Primary outcome measured: Self-confidence<br>Secondary outcomes measured:<br>(1) Client Satisfaction; (2) Drug Attitude; (3) Medication side effects; (4) Positive and negative syndromes; (5) Medication Adherence<br><br>The Morisky scale was used to measure self-reported medication adherence.
**Risk of bias**		
**Bias**	**Authors' judgement**	**Support for judgement**
Random sequence generation (selection bias)	Low risk	Cluster randomisation based on an internet randomisation service (sealed envelope) using block size.
Allocation concealment (selection bias)	Low risk	The allocation list was held by an independent administrator.
Blinding of participants and personnel (performance bias) all outcomes	High risk	Participants and health professionals were not blinded due to the nature of the intervention and the data collection.
Blinding of outcome assessment (detection bias) all outcomes	High risk	The data collection was not blinded due to the fact there was only one principal researcher assigned to the study.
Incomplete outcome data (attrition bias) all outcomes	Low risk	Missing outcome data balanced in numbers across groups with similar reasons for missing data.
Selective reporting (reporting bias)	Low risk	Statistical analyses were conducted blind.
Other bias	High risk	The risk may be explained by limited follow-up. Self-reported responses can be affected by desirability biases.
Montes (2012) [59]		
Methods		Study design: RCT<br>Methods of randomisation: Group assignment was based on a 1:1

	randomisation scheme.<br>Follow-up: Baseline, 3 and 6 months post intervention.<br>Setting: Psychiatric Centres in Spain.<br>Date it was conducted: April 2009 – February 2010<br>Source of funding: AstraZenca Spain.<br>Conflict of interest: Two authors are employees of AstraZenca Spain.	
Participants	Inclusion/exclusion criteria:<br>Patients were included if they were aged between 18 and 65 years, a diagnosis of schizophrenia, clinically stable in the last six months, a single oral antipsychotic medication, follow-up as an outpatient, at least one affirmative answer (indicating suboptimal medication adherence) to the Morisky Adherence Questionnaire and availability of a cell phone capable of receiving SMS messages.<br>Those patients receiving long-acting injectable antipsychotic treatment were excluded.<br>Sample size: 254 (IG=100, TAU=154).<br>Gender: In the intervention group were 65 (65%) men and in the control group 104 (67.5%).<br>Age: The mean age was 38.6 years (SD 10.2) in the intervention group and 40.6 (SD 11.5) in control group.	
Interventions	Type of intervention: Behavioural.<br>Participants assigned to the intervention received daily SMS reminders on their cell phones to take their medication for three months.	
Outcomes	Primary outcome measured: Adherence.<br>The Medication Adherence Questionnaire was used to measure self-reported medication adherence.	
**Risk of bias**		
**Bias**	**Authors' judgement**	**Support for judgement**
Random sequence generation (selection bias)	Low risk	Group assignment was based on a 1:1 randomisation scheme. Randomisation codes were computer-generated by statistician and sealed in envelopes labelled with consecutive numbers, envelopes were opened by the investigator in an ascending order and patients were allocated to intervention and control groups.
Allocation concealment (selection bias)	High risk	Open labelled study.
Blinding of participants and personnel (performance bias) all outcomes	High risk	Open labelled study.
Blinding of outcome assessment (detection bias) all outcomes	High risk	Open labelled study.
Incomplete outcome data (attrition bias) all outcomes	Low risk	Missing outcome data balanced in numbers across groups with similar reasons for missing data.
Selective reporting (reporting bias)	Low risk	The study protocol is not available but it is clear that the published reports include all expected outcomes.
Other bias	Low risk	Self-reported responses can be affected by desirability biases.

Pakpour (2017) [50]

Methods	Study design: RCT<br>Methods of randomisation: Randomisation was done using a computer-generated method.<br>Follow-up: Baseline and 6 months post intervention.<br>Setting: Ten academic centres in Iran.<br>Date it was conducted: September 2014 – October 2016.<br>Source of funding: Not reported.<br>Conflict of interest: Not reported.	
Participants	Inclusion/exclusion criteria:<br>Patients were included if they were 18 years or older, a diagnosis of Bipolar disorder I or II, being treated with a mood stabiliser and were not attending weekly or biweekly psychotherapy.<br><br>Patients were excluded if they had a diagnosis of drug or alcohol misuse disorders, showed evidence of severe borderline personality, needed to change the type and/or the dose of a mood stabiliser, were pregnant or planned to be pregnant in the next year, had any organic cerebral cause for bipolar disorder or had an intellectual disability.<br>Sample size: 270 (IG=134, TAU=136).<br>Gender: In the intervention group were 60 (45%) men and in the control group 67 (49%).<br>Age: The mean age was 41.8 years (SD 8.4) in the intervention group and 41.2 (SD 6.4) in control group.	
Interventions	Type of intervention: Mixed.<br>The multifaceted intervention included two components: psychoeducation for the participants and their family members and motivational interviewing.	
Outcomes	Primary outcome measured: Adherence.<br>The Medication Adherence Rating Scale was used to measure self-reported medication adherence. Adherence was also assessed using objective indices like plasma levels of mood stabilisers.<br>Secondary outcomes measured:<br>(1) Serum levels of mood stabilizers; (2) Clinical symptoms; (3) Quality of life; (4) Measures of intention; (5) Beliefs about medicine; (6) Perceived behavioural control; (7) Automaticity; (8) Action and coping planning; (9) Adverse reactions	
**Risk of bias**		
**Bias**	**Authors' judgement**	**Support for judgement**
Random sequence generation (selection bias)	Low risk	Computer-generated randomisation was reported.
Allocation concealment (selection bias)	Low risk	Assessors, psychologists and psychiatrists were blind to the intervention status of the participants.
Blinding of participants and personnel (performance bias) all outcomes	Low risk	Assessors, psychologists and psychiatrists were blind to the intervention status of the participants.
Blinding of outcome assessment (detection bias) all outcomes	Unclear risk	Assessors, psychologists and psychiatrists were blind to the intervention status of the participants.
Incomplete outcome data (attrition bias) all outcomes	Low risk	Missing outcome data balanced in numbers across groups with similar reasons for missing data.

Selective reporting (reporting bias)	Low risk	The study protocol is not available but it is clear that the published reports include all expected outcomes.
Other bias	Low risk	The study seems to be free of other sources of bias.
Sajatovic (2018) [64]		
Methods		Study design: RCT<br>Methods of randomisation: Block randomisation.<br>Follow-up: Baseline, 10 weeks, 14 weeks and 6 months.<br>Setting: National Institute of Mental Health, U.S.A.<br>Date it was conducted: October 2012 – July 2017.<br>Source of funding: This study was supported by a grant from the National Institute of Mental Health (NIMH) grant NIMH (PI Sajatovic) and by the Clinical and Translational Science Award (CTSC)<br>Conflict of interest: Dr. Sajatovic has research grants from Alkermes, Pfizer, Merck, Janssen, Reuter Foundation, Woodruff Foundation, Reinberger Foundation, National Institute of Health (NIH), and the Centers for Disease Control and Prevention (CDC). Dr. Sajatovic is a consultant to Bracket, Otsuka, Supernus, Neurocrine, Health Analytics and Sunovion and has received royalties from Springer Press, Johns Hopkins University Press, Oxford Press, and UpToDate.
Participants		Inclusion/exclusion criteria: The inclusion criteria were either type I or type II Bipolar disorder as confirmed by the Structured Clinical Interview for DSM-IV Axis I Disorders (SCID), Bipolar disorder for at least two years, prescribed at least one evidence-based Bipolar disorder medication (i.e. lithium, anticonvulsant, or antipsychotic) for at least six months and ≥ 20% non-adherent as assessed by the TRQ. Only individuals unable to participate in study procedures, unable to provide informed consent, and at high risk of harm to self or others were excluded.<br>Sample size: 184 (Mixed intervention group=92, Education group=92).<br>Gender: In the education group were 36% men and in the mixed intervention group 27%.<br>Age: The mean age was 46 years (SD 10.9) in the education group and 49 (SD 9.8) in the mixed intervention group.
Interventions		Type of intervention: Educational versus mixed intervention<br>The education group had five in-person sessions. Four core sessions were followed by one "booster" session and one phone call between the core and booster sessions. Education addresses bipolar disorder treatment broadly including diagnosis and management, and allows time for questions and therapist interaction as needed.<br>The mixed intervention includes an educational and behavioural approach. These modules are psychoeducation focused on the role of medication in bipolar disorder management, Modified Motivational Enhancement Therapy (MET) to address non- adherence related to substance use, Communication with Providers to facilitate appropriate treatment expectations and optimize side effect management, and Medication Routines intended to incorporate medication-taking into lifestyle. Mixed intervention participants had a core series of up to four in-person one-to-one sessions spaced about one week apart over a four–six week period, and one "booster" session four weeks after the core sessions. There was one follow-up phone call between core session completion and the booster session.
Outcomes		Primary outcome measured:<br>(1) Medication adherence; (2) Bipolar disorders symptoms.<br>Adherence was assessed using the Tablets Routine Questionnaire (TRQ), which derives a proportion (%) of days with missed medication doses in

	the last week and last month. TRQ scores ranges from perfect adherence (0% missed) to missing all medication (100% missed). The Medication Event Monitoring System (MEMS) supplemented the TRQ.<br>Secondary outcome measured:<br>(1) Depression; (2) Mania; (3) Clinical symptoms.	
**Risk of bias**		
**Bias**	**Authors' judgement**	**Support for judgement**
Random sequence generation (selection bias)	Unclear risk	Specific details on random sequence generation were missing.
Allocation concealment (selection bias)	Unclear risk	No information on concealment was reported.
Blinding of participants and personnel (performance bias) all outcomes	Unclear risk	No information on blinding of participants and personnel was reported.
Blinding of outcome assessment (detection bias) all outcomes	Unclear risk	No information on blinding of outcome assessors was reported.
Incomplete outcome data (attrition bias) all outcomes	Unclear risk	After randomisation initially 184 enrolled patients and only 148 completed the evaluation.
Selective reporting (reporting bias)	Low risk	The study protocol is not available but it is clear that the published reports include all expected outcomes.
Other bias	Low risk	The study seems to be free of other sources of bias.
Schirmer (2015) [60]		
Methods	Study design: RCT<br>Methods of randomisation: Randomisation was done using a computer-generated method.<br>Follow-up: 1 month.<br>Setting: Centres for Psychiatry in Zwiefalten and Weissenau and the Clinic for Psychiatry in Reutelingen in the south of Germany.<br>Date it was conducted: October 2008 – September 2010.<br>Source of funding: Centre for Psychiatry, South-Württemberg.<br>Conflict of interest: Not reported.	
Participants	Inclusion/exclusion criteria: Inclusion criteria were voluntary, written informed consent, diagnosis of schizophrenia or schizoaffective disorder, age 18-60 years, reachability for home visits, no earlier participation in such a training program and outpatient visits for antipsychotic maintenance treatment after discharge. Exclusion criteria were admission for crisis intervention, absence of written informed consent, high probability that support would be needed for medication intake over a longer period of time and monotherapy with depot antipsychotics.<br>Sample size: 102 (IG=52, TAU=50).<br>Gender: In the intervention group were 27 (52%) men and in the control group 23 (44%).<br>Age: The mean age was 49.8 years in the intervention group and 40.4 in control group.	
Interventions	Type of intervention: Mixed.<br>The training program is conducted in one-to-one lessons with skilled nurses. Participants should learn to prepare their medication by themselves during the hospital stay in the same way they are expected to do it autonomously after discharge. The participants are informed using an	

	educational approach: colour, shape and name of the medication. Level 1 focuses on the scheduled intake of medication, level 2 covers the arrangement of the next day's medication coached by a nurse, in level 3 the dispenser is located in the patient's room and the next day's in level 4, the patient arranges the medication for one week in a dispenser which remains in de patient's room in a locked cupboard. The training takes place in a low-stimulus room in one-to-one lessons.	
Outcomes	Primary outcome measured: Adherence.<br>Three strategies were chosen for this study; pill count, serum levels of the antipsychotic medication and self-reported of medication intake (unclear assessment tool).	
**Risk of bias**		
**Bias**	**Authors' judgement**	**Support for judgement**
Random sequence generation (selection bias)	Low risk	Computer-generated randomisation was reported.
Allocation concealment (selection bias)	Low risk	The clinician who rated the adherence by means of serum levels of antipsychotics was blinded to allocation.
Blinding of participants and personnel (performance bias) all outcomes	Low risk	The study workers who conducted the interviews were blinded with regard to the intervention and to ensure consistency.
Blinding of outcome assessment (detection bias) all outcomes	Low risk	The assessors were blinded.
Incomplete outcome data (attrition bias) all outcomes	High risk	Missing outcome data were reported and were likely to be related to true outcome. Initially 141 enrolled patients, only 102 completed the evaluation.
Selective reporting (reporting bias)	Low risk	The study protocol is not available but it is clear that the published reports include all expected outcomes.
Other bias	Low risk	The risks may be explained by limited follow-up.
Valenstein (2009) [62]		
Methods	Study design: RCT<br>Methods of randomisation: Block randomisation.<br>Follow-up: 6 and 12 months.<br>Setting: Four Departments of Veterans Affairs, Detroit.<br>Date it was conducted: November 2002 – September 2005.<br>Source of funding: Department of Veterans Affairs Health Services Research and Development.<br>Conflict of interest: Not reported.	
Participants	Inclusion/exclusion criteria: The inclusion criteria were having clinical diagnoses of schizophrenia, schizoaffective or bipolar disorder, a treatment plan that included long-term antipsychotic treatment, antipsychotic medication possession ratios of <0.8 in the prior 12 months.<br>Sample size: 118 (IG=58, TAU=60).<br>Gender: In the intervention group were 98% men and in the control group 95%.<br>Age: The mean age was 49.6 years (SD 11.0) in the intervention group and 50.2 (SD 11.7) in control group.	
Interventions	Type of intervention: Educational.<br>The Meds-Help intervention consisted of unit-of-use packaging that	

	included all patient's medications for psychiatric and general medical conditions, a medication and packaging education session, refill reminders mailed two weeks before scheduled refill dates and notification of clinicians when participants failed to fill antipsychotic prescriptions within seven and 10 days of a fill date. The medication education session was conducted by a pharmacist, usually in-person but occasionally by telephone. During this session, the pharmacist reviewed participants' prescribed medications, including treatment indications. The pharmacist also explained unit-of-use medication packaging and plans for interim use of pill boxes when medication changes were made by clinicians before the next shipment of medication packages.	
Outcomes	Primary outcome measured: Adherence.<br>Medication adherence was measured by the Medication Possession Ratios (MPR). A more stringent Composite Adherence Measure (CAM) was also assessed. The MPR is the ratio of number of outpatient day's supply of medication that a patient has received during the designated time period divided by the number of day's supply they needed to receive to take their prescribed dose of antipsychotic continuously during non instutionalised days. The MPR was based on data (pharmacy fills). Participants were considered adherent on the CAM only if their MPR during the study time periods was ≥ 0.8, they reported they "always" took their antipsychotics or only missed antipsychotics "a couple of times" in response to questions from Schizophrenia Outcome Module and their blood test indicated the presence of some antipsychotic medication.<br>Secondary outcome measured:<br>(1) Psychiatric symptoms; (2) Quality of life; (3) Care satisfaction.	
**Risk of bias**		
**Bias**	**Authors' judgement**	**Support for judgement**
Random sequence generation (selection bias)	Low risk	Blocked randomisation scheme by site based on patient's level of adherence in the prior 12 months.
Allocation concealment (selection bias)	Unclear risk	No information on concealment was reported.
Blinding of participants and personnel (performance bias) all outcomes	High risk	Patients could not be blinded to study assignment and research associated were also not blinded due to the costs and logistics of hiring blinded assessors for each site and the likelihood that assessors would be unblended by patient comments
Blinding of outcome assessment (detection bias) all outcomes	High risk	The assessors were not blinded.
Incomplete outcome data (attrition bias) all outcomes	Low risk	Missing outcome data balanced in numbers across groups with similar reasons for missing data.
Selective reporting (reporting bias)	Low risk	The study protocol is not available but it is clear that the published reports include all expected outcomes.
Other bias	Low risk	The risk may be explained by the unclear assessment tool.
Velligan (2013) [63]		
Methods	Study design: RCT<br>Methods of randomisation: Randomisation was done using a computer-generated method.<br>Follow-up: 9 months.	

	Setting: Public mental health clinics in Texas.<br>Date it was conducted: Not reported.<br>Source of funding: National Institutes of Health.<br>Conflict of interest: Not reported.	
Participants	Inclusion/exclusion criteria: Patients were included if they were aged between 18 and 60 years, the diagnosis of schizophrenia, receiving ongoing treatment with an oral antipsychotic, had primary responsibility for taking their own medications, had missed at least one dose of medication in the preceding week, had a stable residence and were able to understand and complete assessments. Patients were excluded if they were on a depot antipsychotic medication, had a hospitalisation in the past three months, had a documented history of significant head trauma, seizure disorder or mental retardation, had a history of substance abuse or dependence in the past month or had a history of violence in the past six months.<br>Sample size: 142 (Med-eMonitor group=48, PharmCAT group=47, TAU=47).<br>Gender: In the Med-eMonitor group were 55% men, in the PharmCAT group 52% and in the control group 53%.<br>Age: The mean age was 43.0 years (SD 10.1) in the Med-eMonitor group, 43 (SD 11.0) in the PharmCAT group and 42 (SD 9.3) in control group.	
Interventions	Type of intervention: Behavioural.<br>The PharmCAT is manual driven and uses environmental supports such as signs, alarms, calendars, checklists and notebooks to record questions for their prescriber, organisation of belonging and pill containers to improve medication adherence. Interventions in PharmCAT are individualised based. Participants in PharmCAT were seen once weekly in their home for 30 minutes.<br>Med-eMonitor treatment consists of a therapist programming prescribing information into the device, setting up the device in the home to fit into the patient's routine (eg, set alarm to take medication, place in a location where he/she is likely to hear the alarm), assisting the patient in accurately filling the device, training the patient how to use the device and providing ongoing trouble shooting. Every three days the therapist was required to check the secure website to determine whether medication was being taken as prescribed and intervene by telephone if patient was missing doses.	
Outcomes	Primary outcome measured: Adherence.<br>Two objective measures of medication adherence were obtained: electronic monitor (opening of pill container) and pill counts.	
**Risk of bias**		
**Bias**	**Authors' judgement**	**Support for judgement**
Random sequence generation (selection bias)	Low risk	Computer-generated randomisation was reported.
Allocation concealment (selection bias)	Unclear risk	No information on concealment was reported.
Blinding of participants and personnel (performance bias) all outcomes	Unclear risk	No information on blinding was reported.
Blinding of outcome assessment (detection bias) all outcomes	Unclear risk	No information on blinding was reported.

Incomplete outcome data (attrition bias) all outcomes	High risk	Fourteen patients (30%) dropped out from the Med-eMonitor intervention.
Selective reporting (reporting bias)	Low risk	The study protocol is not available but it is clear that the published reports include all expected outcomes.
Other bias	Low risk	The risk may be explained by the unclear follow-up.
